# Nomogram for Predicting Early Mortality after Umbilical Cord Blood Transplantation in Children with Inborn Errors of Immunity

**DOI:** 10.1007/s10875-023-01505-8

**Published:** 2023-05-08

**Authors:** Ping Wang, Chao Liu, Zhongling Wei, Wenjin Jiang, Hua Sun, Yuhuan Wang, Jia Hou, Jinqiao Sun, Ying Huang, Hongsheng Wang, Yao Wang, Xinjun He, Xiaochuan Wang, Xiaowen Qian, Xiaowen Zhai

**Affiliations:** 1 Department of Hematology/Oncology, Children’s Hospital of Fudan University, National Children’s Medical Center, Shanghai, 201102 China; 2 Yidu Cloud Technology Inc, Beijing, 100083 China; 3 Nanjing YiGenCloud Institute, Nanjing, 211899 China; 4 Department of Gastroenterology, Children’s Hospital of Fudan University, National Children’s Medical Center, Shanghai, 201102 China; 5 Department of Clinical Immunology, Children’s Hospital of Fudan University, National Children’s Medical Center, Shanghai, 201102 China

**Keywords:** Inborn errors of immunity, nomogram, umbilical cord blood transplantation, pediatric patients

## Abstract

**Purpose:**

Pediatric patients with inborn errors of immunity (IEI) undergoing umbilical cord blood transplantation (UCBT) are at risk of early mortality. Our aim was to develop and validate a prediction model for early mortality after UCBT in pediatric IEI patients based on pretransplant factors.

**Methods:**

Data from 230 pediatric IEI patients who received their first UCBT between 2014 and 2021 at a single center were analyzed retrospectively. Data from 2014–2019 and 2020–2021 were used as training and validation sets, respectively. The primary outcome of interest was early mortality. Machine learning algorithms were used to identify risk factors associated with early mortality and to build predictive models. The model with the best performance was visualized using a nomogram. Discriminative ability was measured using the area under the curve (AUC) and decision curve analysis.

**Results:**

Fifty days was determined as the cutoff for distinguishing early mortality in pediatric IEI patients undergoing UCBT. Of the 230 patients, 43 (18.7%) suffered early mortality. Multivariate logistic regression with pretransplant albumin, CD4 (absolute count), elevated C-reactive protein, and medical history of sepsis showed good discriminant AUC values of 0.7385 (95% CI, 0.5824–0.8945) and 0.827 (95% CI, 0.7409–0.9132) in predicting early mortality in the validation and training sets, respectively. The sensitivity and specificity were 0.5385 and 0.8154 for validation and 0.7667 and 0.7705 for training, respectively. The final model yielded net benefits across a reasonable range of risk thresholds.

**Conclusion:**

The developed nomogram can predict early mortality in pediatric IEI patients undergoing UCBT.

**Supplementary Information:**

The online version contains supplementary material available at 10.1007/s10875-023-01505-8.

## Introduction

Human inborn errors of immunity (IEI), also known as primary immunodeficiencies, are a group of rare diseases caused by monogenic germline mutations. They can be life-threatening if appropriate medical treatment is not provided. Allogeneic hematopoietic stem cell transplantation (allo-HSCT) offers life-saving and curative treatment for the vast majority of IEI [1-5]. Although a human leukocyte antigen (HLA)-matched unaffected sibling is the preferred donor, it is available for only less than one-third of patients [6, 7]. Unrelated umbilical cord blood (UCB), as an important alternative stem cell source, is a suitable option for patients for whom a suitable donor is not available in due time [8]. However, transplant-related mortality (TRM) is a major obstacle in unrelated umbilical cord blood transplantation (UCBT).

Mortality after allo-HSCT can be classified into very early, early, intermediate, and late mortality, based on the time between HSCT and death [9]. The exact definitions of these classifications vary by transplant indication (e.g., malignancy vs. non-malignancy) and transplant characteristics (e.g., stem cell source). Early all-cause mortality is of great interest in patients undergoing UCBT for several reasons. First, mortality in patients with IEI occurs mainly in the early stage after UCBT, due to delayed engraftment, graft failure or poor graft function, acute graft-versus-host-disease (GvHD), and infection [3-5, 10]. Second, although the overall mortality after UCBT in children has decreased in recent years, the trend in early all-cause mortality has not been reported [7, 9]. Third, patients at high risk of early mortality must be identified for clinicians and patient guardians to make informed decisions.

Despite its importance, early mortality after UCBT in IEI patients has not been well-studied for several reasons. First, IEI are "very rare" and highly heterogeneous. As a result, it is difficult to obtain adequate sample size for IEI subgroup analysis. Second, analytic tools (e.g., machine learning algorithms) have rarely been used to reveal underlying patterns between risk factors and outcomes of interest (i.e., early all-cause mortality) in the landscape of hematology or transplantation. Lastly, the threshold for early mortality after UCBT, defined as a day post-transplant, when mortality transitions from a designation of "early" to “intermediate”, remains undetermined [9, 11-13].

Machine learning is a data-driven analytic approach [14] to establishing a model by recognizing the underlying patterns [15], including the thresholds for specific variables. Machine learning is likely to be increasingly integrated into the research and practice landscape of hematology [16]. However, machine learning is yet to be applied in identifying risk factors for early mortality after allo-HSCT and predicting early all-cause mortality in pediatric IEI patients.

Therefore, in this study, we first established a cutoff value for distinguishing different phases concerning mortality (e.g., early mortality) based on a data-driven approach and real-world evidence in pediatric IEI patients who underwent UCBT and developed and validated a prediction model for early all-cause mortality after UCBT based on pretransplant factors.

## Materials and Methods

### Patients

Patients included in this study were diagnosed with IEI according to the criteria proposed by the International Union of Immunological Societies, which include: typical clinical manifestations, monogenic mutations, and/or functional identifications [17]. All patients were between 0 and 14 years old and had undergone UCBT at the Children’s Hospital of Fudan University between February 2014 and December 2021. They all met the HSCT guidelines for IEI [18, 19] and had no matched-related donor. No exclusion criteria were set for this study.

This study was approved by the ethics board of the Children’s Hospital of Fudan University. Written informed consent was obtained from the guardians of all the patients.

### Transplantation-related definitions

Conditioning regimens were classified as myeloablative conditioning with busulfan (BU) >8 mg/kg in combination with cyclophosphamide (100 mg/kg) and/or fludarabine (150–175 mg/m^2^) and reduced-intensity conditioning with BU ≤8 mg/kg in combination with cyclophosphamide and/or fludarabine. The BU dosage for patients with severe combined immunodeficiency (SCID), very early onset inflammatory bowel disease (VEO-IBD), and chronic granulomatous disease (CGD) was (6.4–13.2), (8.0–14.4), and (12.0–19.2) mg/kg, respectively. GvHD prophylaxis mainly consisted of calcineurin inhibitors (cyclosporin A or tacrolimus) alone or in combination with mycophenolate mofetil. HLA compatibility was determined by high-resolution typing for HLA-A, -B, -C, -DR, and -DQ loci.

### Characteristics and outcomes

Patient (e.g., demographics, clinical manifestations, medical history, laboratory results, and diagnosis), donor (stem cell count and HLA compatibility), and follow-up information (e.g., complications, survival status, and cause of death) were collected retrospectively using a standardized, computerized form.

Serum biochemical indices (albumin [Alb], alanine aminotransferase [ALT], total bilirubin, and immunoglobulin), inflammatory biomarkers (procalcitonin [PCT], ferritin, and Interleukin 6 [IL-6]), and lymphocyte subsets were measured approximately one week before the start of conditioning. C-reactive protein (CRP) was measured just before the initiation of conditioning. The elements in the medical history category (e.g., intestinal infection, severe pneumonia) are defined in detail in Table S1.

With the exception of the outcomes of interest (early mortality and causes of death), all the aforementioned analysis variables were pretransplant factors. A complete list of the variables is shown in Table S2.

We plotted the cumulative density plot of the interval between UCBT and the last follow-up or death. Early mortality was defined based on the pattern depicted in the plot using the ‘elbow’ method [20].

### Modeling

When preparing the data for modeling, we first checked for missing patterns in the variables. Missing values were then handled with a multivariate imputation strategy to prepare the data matrix for modeling, and one of the five imputed data sets was randomly selected. Variables used in the analyses were converted to numeric or binary values (e.g., 1 indicates male, 0 indicates female). Using one-hot encoding method [21], we converted IEI disease diagnosis (categorical data) to multidimensional binary vectors. The primary outcome variable was converted to 0 (negative) or 1 (positive) indicating early mortality.

The cohort dataset was divided based on a marker time point. Data from patients who underwent UCBT before and after January 1, 2020, were assigned to a training or validation set, respectively, before feature selection and model development. We performed ensemble feature selection to alleviate and compensate for specific biases associated with single feature selection and to increase the stability of feature selection based on a different training set [22].

Machine learning algorithms were used to develop a prediction model. Multivariate logistic regression (LR), Lasso [23], random forest, and extreme gradient boosting (XGBoost) were used for the selected variable and all-variable sets, as appropriate. Subsequently, the optimal parameters of the machine learning algorithms were obtained through cross-validation using the training set. Finally, the performance of the model was compared using the validation set. Among all the models, the LR with the selected variables provided the highest area under the curve (AUC) using the validation set.

The resulting model was further validated using the bootstrap method (1000 times) after a performance assessment in the validation set [24]. Finally, a nomogram was developed to visualize the model.

### Model evaluation

Performance of the best model (i.e., LR with the selected variables) was evaluated using the AUC. The accuracy of the optimal cutoff value was assessed using sensitivity, specificity, and positive and negative predictive values (PPV and NPV, respectively).

Patients in the temporal validation cohort were classified into two prognostic groups (i.e., high-risk group vs. low-risk group) based on their predicted probability of early mortality and the selected cutoff probability, and their survival curves were compared using the Kaplan–Meier method. In addition, a decision curve analysis (DCA) was performed to evaluate the potential clinical benefits of using the model to identify patients at high risk for early mortality. DCA [25] evaluates the value of a predictive model when making clinical decisions. Three strategies were compared: selecting all patients for intervention (i.e., treating all), selecting no patients (i.e., treating none), and selecting patients based on the nomogram.

### Statistical analyses

Unpaired, two-tailed *t*-test and Wilcoxon test were used to compare the distribution of continuous variables. For variables that did not follow a normal distribution, the median and quantile values were compared. The chi-square test was used to quantify the relationship between categorical variables (e.g., label balance between the training and validation sets). The Multivariate Imputation by Chained Equations (MICE) package was used for missing value imputation. The roc.test() function in the ‘pROC’ package was used to compare the two Receiver Operator Characteristic (ROC) curves on the training and validation sets. Statistical significance was set at p = 0.05. Statistical analyses were performed using R version 4.0.1 (R Foundation for Statistical Computing, Vienna, Austria).

## Results

### Patient characteristics

**Fig. 1:**
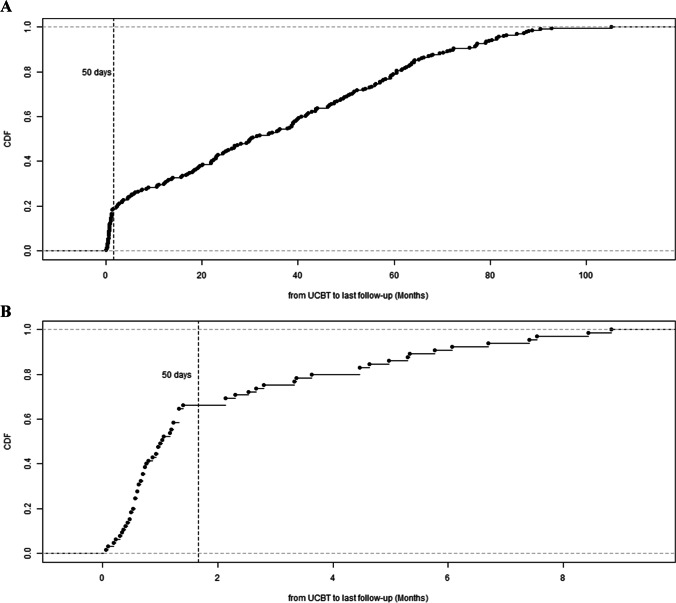
Cumulative density plot of interval between transplantation and the last follow up. **A**. All patients enrolled in this study (N = 230). **B**. Patients with mortality in this study (N = 65)

Data from the 230 patients (181 male and 49 female) who underwent UCBT between February 2014 and December 2021 were included in this study. Patient demographics and UCBT characteristics are shown in Table 1. The median age at UCBT was 14.50 months (interquartile range [IQR], 8.71–29.18 months). Sixty-seven patients were diagnosed with CGD, 73 with VEO-IBD, 48 with SCID, and 42 with other types of IEI, including Wiskott–Aldrich syndrome (WAS), leukocyte adhesion deficiency (LAD), and hyper-IgM (HIgM) syndrome. The genetics of the diagnoses are detailed in Table S3. The engraftment rate was 81.7% (188/230). The median follow-up time was 30.17 months (IQR, 5.44–56.78 months) after UCBT. During follow up, 65 patients (28.3%) died after UCBT, resulting in an overall survival rate of 71.7% (Fig. S1). Early mortality was defined as death within 50 days after UCBT (Fig. 1). Of the 230 patients, 43 (18.7%) had early mortality due to infection (29/43), organ dysfunction (11/43), or acute GvHD (3/43).

**Table 1:** Overview of 230 patients with inborn errors of immunity (IEI) who underwent unrelated umbilical cord blood transplantation (UCBT)

Characteristics	Total (N = 230)
Demographics	
Sex	
Female	49 (21.3%)
Male	181 (78.7%)
Weight (kg)	9 (7–11)
Height (cm)	73 (67–84)
Disease	
CGD	67 (29.1%)
SCID	48 (20.9%)
VEO-IBD	73 (31.7%)
Other IEI*	42 (18.3%)
Age at onset (days)	23 (10–116)
Age at diagnosis (months)	7.67 (3.50–17.73)
Age at UCBT (months)	14.50 (8.71–29.18)
Length of follow-up (months)	30.17 (5.44–56.78)
UCBT characteristics	
CD34 (×10^5^/kg)	3.36 (2.20–5.32)
TNC (×10^7^/kg)	12.71 (8.75–16.16)
HLA Compatibility (/10)	8 (8–9)
Conditioning regimen	
Reduced-intensity conditioning (BU≤8mg)	
BU/CY/FLU	11 (4.8%)
BU/CY/FLU/ATG	1 (0.4%)
BU/FLU	1 (0.4%)
Myeloablative conditioning (BU>8mg)	
BU/CY/FLU	138 (60.0%)
BU/CY/FLU/ATG	38 (16.5%)
BU/CY	39 (17.0%)
BU/FLU	1 (0.4%)
BU alone	1 (0.4%)
GvHD prophylaxis	
CNI	183 (79.7%)
CNI+MMF	45 (19.6%)
CNI+ steroid	2 (0.9%)
Acute GvHD	
No GvHD	94 (40.9%)
Grade I	49 (21.3%)
Grade II	45 (19.6%)
Grade III	15 (6.5%)
Grade IV	27 (11.7%)
Chronic GvHD	23 (10.0%)

Continuous variables are presented as median (interquartile range), and categorical variables are presented as counts (percentages)

^*^Other IEI includes CARD11 deficiency(1/42), CD25 deficiency (1/42), chronic infantile neurologic cutaneous and articular syndrome (1/42), hyper IgE syndrome (4/42), hyper IgM syndrome (9/42), immune dysregulation, polyendocrinopathy, enteropathy X-linked (2/42), leukocyte adhesion deficiency type I (5/42), LRBA deficiency (3/42), severe congenital neutropenia (1/42), STAT5B deficiency (1/42), TRNT1 deficiency (1/42), Wiskott-Aldrich Syndrome (9/42), X-linked lymphoproliferative disease (3/42), ZAP-70 deficiency (1/42)

ATG anti-thymocyte globulin, BU busulfan, CGD chronic granulomatous disease, CNI calcineurin inhibitors, CY cyclophosphamide, FLU fludarabine, GvHD graft versus host disease, HLA human leucocyte antigen, MMF mycophenolate mofetil, SCID severe combined immunodeficiency, TNC total nucleated cells, UCBT umbilical cord blood transplantation, VEO-IBD very early onset inflammatory bowel disease

A comparison between the early mortality group and other patients group (died after day 50 or survived) is presented in Table 2. The serum albumin, CD4-positive lymphocytes (absolute count and ratio), CD19-positive lymphocytes (absolute count), and CD3-positive lymphocytes (absolute count) were significantly lower in the early mortality patients than in intermediate to late mortality patients and survivors. Patients with early mortality showed a significant positive correlation with elevated CRP (CRP >= 8 mg/L), higher IL-6, a medical history of sepsis, and intestinal infection. Early mortality was significantly lower in patients with CGD. Insignificant differences were found in other variables between the different mortality groups. A comparison of clinical variables between the training and validation sets is provided in Table S2.

**Table 2:** Demographic, clinical, and biological characteristics of early mortality and other patients cohorts

Characteristics	Early Mortality (N=43)	Other Patients* (N=187)	*p*-value
Demographics			
Sex			0.785
Female	8 (18.6%)	41 (21.9%)	
Male	35 (81.4%)	146 (78.1%)	
Weight (kg)	8.0 (6.5–9.9)	9.0 (7.0–11.5)	0.052
Height (cm)	73 (65–78)	73 (67–85)	0.225
BMI	15.38 (2.08)	15.80 (2.35)	0.285
Disease			0.012
CGD	5 (11.6%)	62 (33.2%)	0.009
SCID	13 (30.2%)	35 (18.7%)	0.142
VEO-IBD	19 (44.2%)	54 (28.9%)	0.078
Other IEI	6 (14.0%)	36 (19.3%)	0.554
Age at onset (days)	31 (11–107)	22 (10–113)	0.657
Age at diagnosis (months)	5.17 (3.32–17.02)	7.80 (3.70–17.70)	0.286
Age at UCBT (months)	15.20 (8.70–27.75)	14.43 (8.80–30.40)	0.587
Onset to diagnosis (months)	3.13 (1.85–7.50)	5.00 (2.15–12.58)	0.170
Diagnosis to UCBT (months)	6.50 (3.05–10.93)	6.10 (3.18–10.70)	0.908
Medical history^#^			
Sepsis	21 (48.8%)	40 (21.4%)	<0.001
Pneumonia	41 (95.3%)	158 (84.5%)	0.103
Pulmonary fungal infection	10 (23.3%)	49 (26.2%)	0.837
Severe pneumonia	5 (11.6%)	10 (5.3%)	0.245
Intestinal infection	38 (88.4%)	125 (66.8%)	0.009
Urinary tract infection	5 (11.6%)	45 (24.1%)	0.115
CNS infection	3 (7.0%)	7 (3.7%)	0.601
SSTI	24 (55.8%)	98 (52.4%)	0.815
CMV infection	4 (9.3%)	28 (15.0%)	0.469
EBV infection	1 (2.3%)	11 (5.9%)	0.572
BCG disease	7 (16.3%)	43 (23.0%)	0.449
Liver dysfunction	21 (48.8%)	66 (35.3%)	0.140
Laboratory tests			
Albumin (g/L)	35.20 (32.17–40.25)	41.07 (37.50–43.10)	<0.001
ALT (U/L)	21.7 (11.5–33.8)	21.8 (13.0–39.5)	0.467
Total bilirubin (μmol/L)	3.9 (3.3–5.9)	4.4 (3.4–5.9)	0.832
IgA (g/L)	0.42 (0.13–0.84)	0.34 (0.15–1.12)	0.977
IgG (g/L)	9.96 (7.05–12.10)	8.85 (6.00–12.30)	0.442
IgM (g/L)	0.81 (0.38–1.59)	0.71 (0.37–1.27)	0.555
IgE (KU/L)	25.47 (9.54–40.36)	25.32 (10.59–103.35)	0.243
CD19 count (/μL)	360.3 (205.1–699.9)	632.2 (310.2–1153.6)	0.005
CD19 ratio (%)	15.52 (8.87–26.89)	21.08 (12.38–31.36)	0.064
CD3 count (/μL)	1676.6 (358.1–2313.9)	1932.1 (1173.9–2890.7)	0.017
CD3 ratio (%)	62.22 (35.50–73.20)	62.46 (51.89–71.37)	0.882
CD4 count (/μL)	871.4 (179.8–1149.1)	1086.2 (652.2–1689.7)	0.005
CD4 ratio (%)	30.93 (12.79–38.73)	34.96 (25.14–42.89)	0.048
CD8 count (/μL)	513.0 (177.0–1021.7)	643.4 (359.3–1052.5)	0.180
CD8 ratio (%)	24.38 (14.07–31.78)	20.31 (13.97–28.79)	0.467
CD56 count (/μL)	268.2 (112.7–443.2)	314.4 (186.2–578.1)	0.212
CD56 ratio (%)	10.44 (6.76–29.48)	10.88 (6.03–17.28)	0.135
elevated CRP (>= 8 mg/L)	20 (46.5%)	33 (17.6%)	<0.001
elevated PCT (>= 0.5 ng/ml)	1 (2.33%)	1 (0.53%)	0.818
IL-6 (imputed) (pg/ml)	31.22 (13.05–105.50)	15.49 (6.33–76.52)	0.008
IL-6 (pg/ml)	31.22 (13.05–105.50)	17.88 (6.33–44.71)	0.016
ferritin (imputed) (ng/ml)	82.54 (29.66–165.85)	67.10 (36.47–133.60)	0.567
ferritin (ng/ml)	82.54 (29.93–160.00)	65.33 (34.88–123.65)	0.426
UCB parameters			
CD34 (×10^5^/kg)	2.84 (2.09–4.25)	3.51 (2.22–5.89)	0.114
TNC (×10^7^/kg)	10.80 (8.09–14.82)	13.01 (9.00–16.71)	0.193
HLA Compatibility (/10)	8 (8–9)	8 (8–9)	0.183

Continuous variables are presented as median (interquartile range), and categorical variables are presented as counts (percentages)

*Other patients: died after day 50 or survived

BMI is the only variable that follows a normal distribution; thus, it is presented as mean (SD)

^#^Since thorough eradication of residual infection in patients with IEI is particularly difficult and there is a lack of uniformity in the definition of ‘active infection’, ‘Medical history’ represents ever had infection or active infection

ALT alanine aminotransferase, BCG Bacillus Calmette-Guérin, BMI body mass index, CGD chronic granulomatous disease, CMV Cytomegalovirus, CNS central nervous system, EBV Epstein–Barr virus, HLA human leucocyte antigen, VEO-IBD very early onset inflammatory bowel disease, SCID severe combined immunodeficiency, SSTI skin/soft tissue infection, TNC total nucleated cells, UCB umbilical cord blood

### Feature selection

Two of the 49 variables had missing values (missing rate IL6: 11/230 4.78%; ferritin: 26/230 11.3%). After missing value imputation, no significant outliers or perfect collinearity were found in the feature set, and the correlation matrix by heatmap is shown in Fig. S2. Next, we calculated the quantitative ensemble importance for each variable in the training set using the ensemble feature selection algorithm (Fig. S3) and all candidate variables were ranked based on their importance. In a step-wise approach, we added features from the top-ranked variables until Akaike information criterion (AIC) of the Linear Regression model fitting would not decrease (Table S4). Four variables (Alb, cd4_abs [pretransplant CD4 count], elevated CRP and sepsis) were selected. We did not select candidate variables based on univariate analysis.

### Development and validation of the early mortality-predicting models

**Fig. 2:**
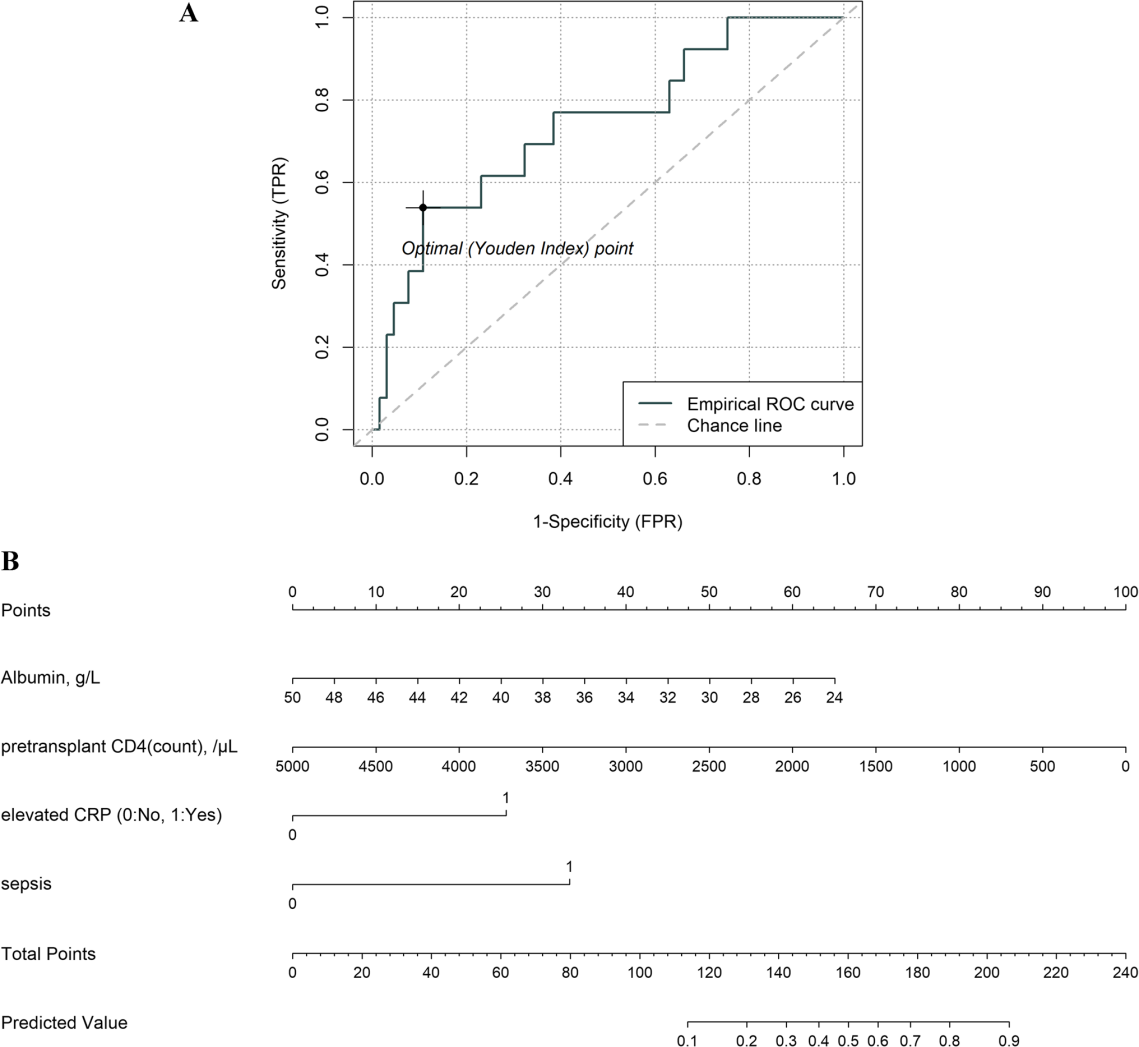

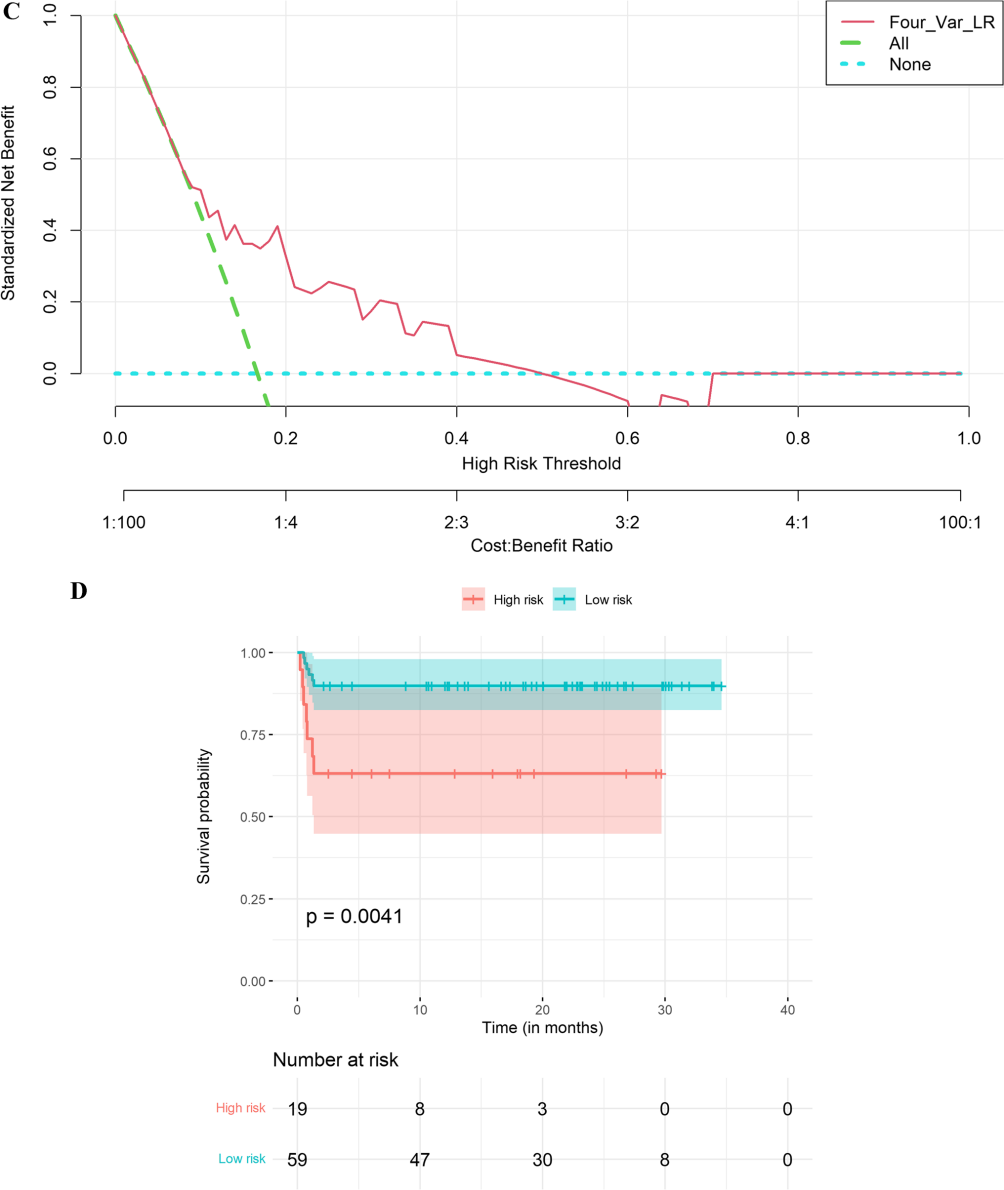
Nomogram for predicting early mortality and model evaluation. **A**. Receiver operating characteristic (ROC) curves for evaluating the model’s discrimination ability in validation. Panel A shows the ROC curve for the Four-Var LR model, the area under curve (AUC) is 0.7385 (95% confidence interval [CI], 0.5824–0.8945). **B**. Nomogram. The nomogram was developed in the derivation cohort with pretransplant albumin (Alb), pretransplant absolute CD4 (cd4_abs), elevated CRP, and medical history of sepsis (sepsis). Instructions on using nomogram: first, find the position of each variable on the corresponding axis; then, draw a line to the points axis for the number of points and add the points from all variables; and finally, draw a line from the total points axis to the lower line of the nomogram for determining the early mortality probabilities. **C**. Decision curve analysis (DCA) in the validation set. **D**. Comparison of survival curves (event: early mortality) in different risk groups by the risk estimation nomogram (p = 0.0041)

There was no statistical difference in early mortality incidence between the training and validation sets (19.74% vs. 16.67%, p = 0.699). The multivariate LR analysis with Alb, cd4_abs, elevatedCRP, and sepsis performed better than the other models in the validation set, with an AUC of 0.7385 (95% CI, 0.5824–0.8945) (Fig. 2A). The bootstrap-corrected AUC was 0.8052 (Table S4). In the validation cohort, the sensitivity, specificity, PPV, and NPV for differentiating early mortality were 0.5385, 0.8154, 0.3684, and 0.8983, respectively (Table 3). A comprehensive comparison of the model performance is presented in Table S5.

**Table 3:** Prediction score accuracy of the nomogram estimating the risk of early mortality after UCBT in pediatric patients with inborn errors of immunity (IEI)

Variable	Value (95%CI)	
	Training Cohort (N = 152)	Validation Cohort (N = 78)
Number of positive labels	30 (19.74%)	13 (16.67%)
AUC	0.827 (0.7409–0.9132)	0.7385 (0.5824–0.8945)*
Cutoff probability	0.2	0.2
Sensitivity (%)	76.67 (57.72–90.07)	53.85 (25.13–80.78)
Specificity (%)	77.05 (68.57–84.18)	81.54 (69.97–90.08)
PPV (%)	45.10 (31.13–59.66)	36.84 (16.29–61.64)
NPV (%)	93.07 (86.24–97.17)	89.83 (79.17–96.18)
Positive likelihood ratio	3.3405 (2.2836–4.8865)	2.9167 (1.4237–5.9750)
Negative likelihood ratio	0.3028 (0.1572–0.5835)	0.5660 (0.3111–1.0298)

*roc.test() for two Receiver Operator Characteristic (ROC) curves p > 0.3

AUC Area under the curve, CI confidence interval, NPV Negative predictive value, PPV Positive predictive value

A nomogram was developed based on the best model (Fig. 2B). The DCA results for the model in the validation set are presented in Fig. 2C. The DCA results suggest that the machine learning-based prediction model had a net benefit compared with the “treat all” and “treat none” strategies for thresholds above 0.1.

Furthermore, a risk stratification based on the early mortality probability of each patient predicted by the nomogram was performed to divide all patients in the temporal validation cohort into two prognostic groups: the high-risk group (19/78, 7 early mortality, predicted probability >= 0.2) and the low-risk group (59/78, 6 early mortality, predicted probability < 0.2) and their survival curves were compared using the Kaplan-Meier method. The results showed that the difference was statistically significant (p = 0.0041) (Fig. 2D).

## Discussion

Owing to the rarity of IEI and the difficulty in predicting the prognosis of transplantation, few studies have comprehensively investigated patients with IEI and early mortality after HSCT. Existing models were developed using patients with malignancy [13, 26, 27]. However, IEI patients are fundamentally different from patients with malignancy. The comorbidity index for hematopoietic cell transplantation has been validated and can distinguish between the risk of death after HSCT for patients with nonmalignant disease [28]. Nevertheless, it was not specific for predicting the prognosis of pediatric patients with IEI who underwent UCBT. Thus, a model that can predict early mortality after UCBT in children with IEI is urgently needed. To the best of our knowledge, this is the first study to develop such a model. Here, we included 230 pediatric patients with IEI who underwent UCBT to demonstrate the pattern of early mortality and proved that early mortality can be predicted using a machine learning approach.

Compared with bone marrow and peripheral blood stem cells, UCB has the advantages of rapid availability, less stringent HLA matching, lower risk of viral infection transmission, more versatile transplant planning, and no risk of donor refusal [29]. UCBT is a suitable option for patients without an available matching (related or unrelated) donor, especially for patients who need an urgent transplant (e.g., patients with SCID) and pediatric patients with low body weight. However, the disadvantages of UCBT, such as slower engraftment, graft failure, delay in immune reconstitution due to limited stem cell dose, and the associated significantly increased risk of infection, should not be underestimated [29]. Specifically, patients with IEI usually face the heavy burden of infection and inflammation. Given the limited cell dose of UCB and the underlying IEI disease, the risk of infection and organ dysfunction (the major cause of early mortality) after transplantation is of particular concern. Thus, there is a need to create and validate a model that can predict early mortality after UCBT in patients with IEI that can help clinicians make better-informed decisions considering the risk-benefit ratio.

In our study, the cumulative density plot of time from transplant to the last follow-up or death in all patients and in the subgroup of patients who died during the follow-up period showed that the majority (43/65, 66.2%) of mortality in pediatric patients with IEI after UCBT occurred within 50 days of transplantation. All-cause mortality is more heterogeneous (in terms of causes of death) than early mortality, which may explain the difficulty in predicting mortality after HSCT. Our results showed that the main causes of early mortality were infections, organ dysfunction, or acute GvHD. We further compared the interval between transplantation and the last follow-up among patients with different causes of death and found insignificant differences (Fig. S4). Given the dominance and relative homogeneity of early mortality, we chose “early mortality” as the outcome of interest.

To date, a consensus on the cutoff point for early mortality has not been reached. Some studies reported the cutoff point as 30–100 days in adult patients [9, 11, 12], and early mortality was defined as death during hospitalization for HSCT [13]. However, in our data, the cumulative density plot of the interval between UCBT and the last follow-up demonstrated a sudden change in the slope at the 50-day cutoff point. Thus, we defined 50 days as a cutoff point to distinguish patients with early mortality from patients who died after 50 days or survivors.

**Fig. 3:**
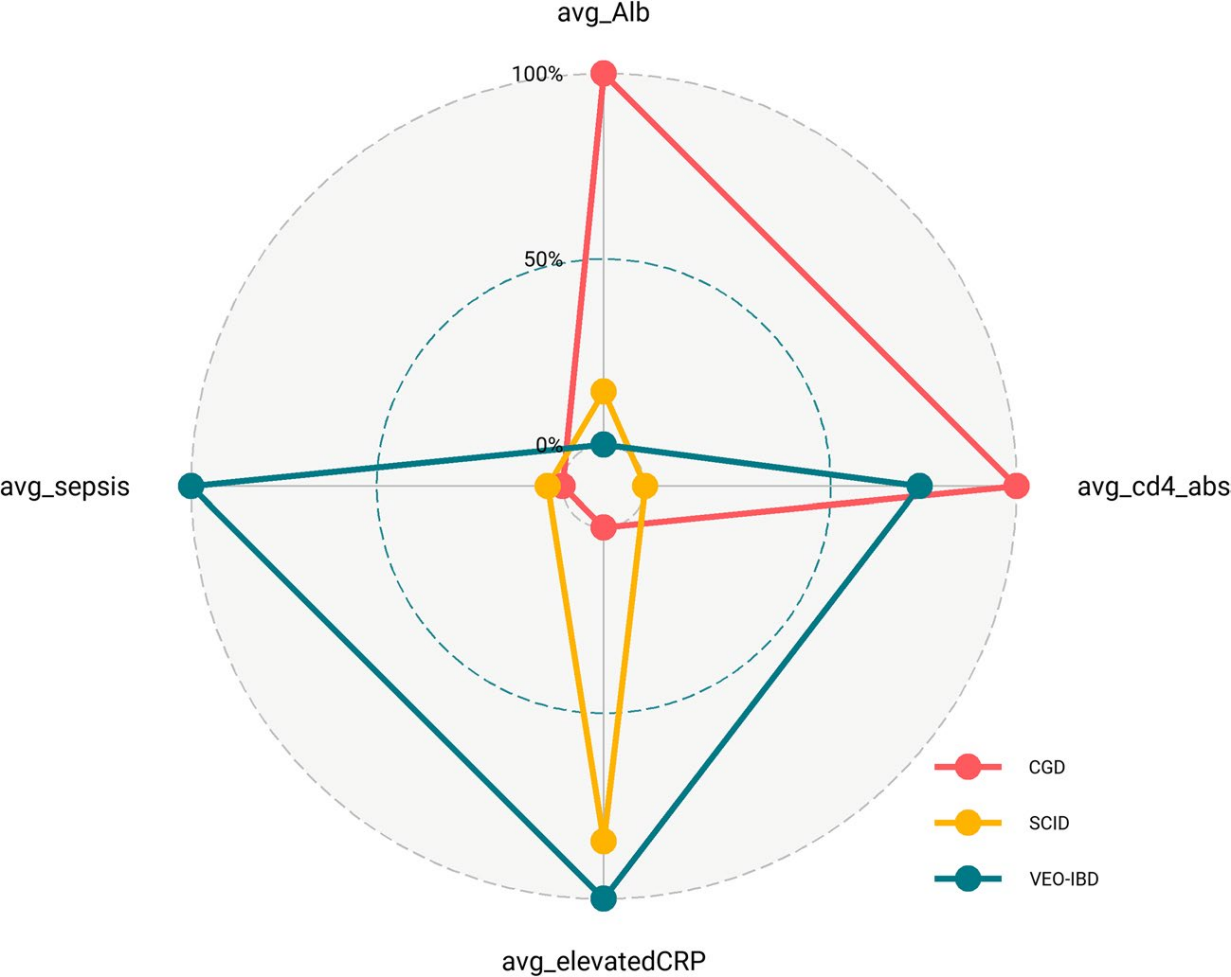
Risk profile for different IEI subtypes represented by the four selected variables. The mean value of each variable was first calculated across patients in each IEI subtype (for binary variables [i.e., sepsis, elevated CRP], mean values equal to its positive rate); each variable has three disease-specific mean values, these summaries are then rescaled to the [0, 1] interval

To account for the heterogeneity of this IEI cohort with multiple disease subtypes, we adopted a one-hot encoding approach, i.e., the presence or absence of a particular IEI subtype considered during modeling. After the modeling process was completed, we attempted to add the disease information (one-hot encoded disease variable) back to the best-performing model and observed that the model performance did not improve in either the training or validation set (Table S6). This indicated that the selected variables were informative and that disease heterogeneity was represented by these selected variables (cd4_abs, Alb, elevatedCRP, and sepsis). To further verify the disease heterogeneity represented by the selected variables, we used the selected variables to depict different disease subtypes; the risk profile in the radar plot indicated that the selected variables constitute a good representation of disease heterogeneity (Fig. 3). Overall, these findings highlight that the prediction model could estimate the risk of early mortality independently of the underlying disease.

In the risk estimation nomogram, pretransplant serum albumin was negatively correlated with the risk of early mortality after UCBT. Serum albumin is a biomarker of nutrition, inflammation, and hepatic synthetic function [30]. Several studies have shown that pretransplant serum albumin is an important prognostic marker of autologous stem cell transplantation and allo-HSCT [31-33]. Patients with IEI are at risk of recurrent infections, inflammatory diseases, growth retardation and failure to thrive resulting in hypoalbuminemia. Furthermore, hypoalbuminemia is associated with poor nutritional status, severe disease burden, repeated exposure to therapeutic drugs, and decreased tolerance to conditioning regimens, which could contribute to early mortality after UCBT. Our study highlights the predictive value of pretransplant serum albumin level in patients with IEI who underwent UCBT. Moreover, serum albumin detection is inexpensive and practical, making it an ideal biomarker for clinical decision-making, such as aggressive nutritional intervention and effective infection control before UCBT.

Infection and inflammation at transplant are critical risk factors that affect HSCT outcomes [34]. Despite optimal intervention (e.g., protective isolation, antibiotics, immunoglobulin replacement, and targeted agents) before transplantation, infection and inflammation cannot be completely relieved at transplantation due to the nature of IEI. In this study, acute phase reactant proteins (e.g., CRP, ferritin) and cytokine (e.g., IL-6), as objective inflammatory biomarkers, were used to reflect the status of infection and inflammation and we found that elevated CRP was a significant risk factor for post-UCBT early mortality in patients with IEI. Since elevated CRP is associated with active inflammation and previous studies showed that control of inflammation activity is the key to successful outcome [34], patients with elevated CRP prior to transplant should proceed with caution or be under intensive monitoring after UCBT.

In our study, a low absolute count of CD4-positive lymphocytes was associated with a high risk of early mortality. CD4-positive lymphocytes play an important role in adaptive immunity. The deficiency of immune defense and surveillance caused by CD4-positive lymphocytopenia can provoke fatal opportunistic infections and lead to malignancy [35, 36]. In patients with IEI, a decrease in CD4-positive lymphocytes is often closely associated with combined immunodeficiency diseases, especially SCID [37-40]. Even when we excluded SCID patients (N = 48) characterized by low CD4 levels from our cohort, the absolute count of CD4-positive lymphocytes in the early mortality group remained significantly lower than that in the other patients group (p = 0.03). Thus, our study highlights the association between lower CD4 levels and early mortality, and clinicians should be alert to patients whose CD4-positive lymphocyte count decrease before UCBT.

Our result also showed that a history of sepsis was positively associated with early mortality after UCBT in patients with IEI. Few studies have shown the association between a history of sepsis and HSCT prognosis [41]. Sepsis is a life-threatening condition caused by a dysregulated host response to infection, and it remains a leading cause of morbidity and mortality in pediatric patients worldwide [42, 43]. Virtually all tissues and organs can undergo dysfunction following sepsis [44]. Although sepsis was under control in our cohort before transplantation, patients with a history of sepsis were more likely to experience early mortality. We speculate that the long-term consequences of sepsis had a negative impact on UCBT prognosis. Our results highlight the need to identify the history of sepsis as a risk factor for an unfavorable prognosis after UCBT in patients with IEI since patients who undergo UCBT before the onset of sepsis may have better outcomes.

CD34-positive cell dose and HLA compatibility are important markers for HSCT outcomes. A sufficient number of CD34-positive stem cells is the key to rapid and durable engraftment of donor cells [45-49]. HLA-locus mismatch increases the risk of graft failure, GvHD, and mortality [50]. However, in the present study, none of these factors was selected by feature selection, and therefore, were omitted from the final model. Furthermore, no significant difference in CD34-positive stem cell dose and HLA compatibility was observed between patients with early mortality and other patients (Table 2). Since all patients included in this study were pediatric, the CD34 counts of this cohort were rather high in nearly all the patients (225/230), thus its impact may not be obvious. Similarly, due to the availability of high-resolution typing and stringent donor selection, HLA typing was a relatively good match (8–9/10) for UCBT, and its impact will also be less important in this setting. Furthermore, the reduced impact can also be attributed to the biological characteristics of UCB - high proliferative capacity and high immune plasticity, which allowed engraftment despite a 1-log lower cell number and a wider HLA disparity between donor and recipient [51, 52]. Moreover, with the improvement of HSCT technology, such as the intensity of the conditioning regimen and GvHD prophylaxis, a limited stem cell count or an HLA-related barrier no longer has an independent impact on the outcomes of patients with IEI.

The findings of the present study have several clinical implications. First, our model yielded a good AUC, indicating a good discriminatory ability. The DCA of the validation set revealed that the model provided the highest overall net benefit when the probability threshold was approximately 0.2. At this threshold probability, sensitivity and specificity were 0.5385 and 0.8154, respectively. Second, the risk estimate provided a net benefit across reasonable probability thresholds (0.1–0.5) using DCA. Clinicians have the flexibility to adjust the threshold for better sensitivity (i.e., a lower risk threshold) or better specificity (i.e., a higher risk threshold) in a case-by-case manner. Third, the nomogram developed in this study can be used to facilitate clinical decision-making and determine the predicted risk to optimize patient treatment or determine the optimal time frame for transplantation. Clinicians can use this model to modify treatment options (e.g., gene therapy and clinical trials). Furthermore, patients and their families can make informed decisions regarding predicted risk, in cases where the risk level of early mortality cannot be altered.

### Limitations

This study had several limitations. First, the analysis was based on patient data from a single center, which may be influenced by selection bias. The selection bias may also induce lack of heterogeneity in some variables, including BU-based conditioning, degree of HLA match, and CD34 count, which are important for transplant outcomes. The lack of heterogeneity limits the utility of these variables in the early mortality risk model. A multicenter study and external validation are needed to confirm our results. Second, due to the long-term nature of the study, we could not find or access the information needed to determine ‘performance status’, so we had to exclude it from the list of potential predictors when designing the study. However, by including variables that can be objectively measured, we ensured the reliability and accuracy of the variables included. Third, the present study was retrospective in nature. A prospective study is required to confirm the usefulness of the nomogram developed in this study. Nonetheless, the large dataset in the analysis, the temporal validation approach, the ensemble feature selection, and the bootstrap validation greatly improved the validity and robustness of the final model.

## Conclusions

Using real-world data from pediatric IEI patients and data-driven analysis, we developed a nomogram that can predict early mortality after UCBT based on four pretransplant risk factors. The nomogram performed well in training and validation sets. The personalized prediction model based on machine learning can improve decision-making and outcomes for patients with IEI who have undergone UCBT.

## Supplementary information

ESM 1 (DOCX 1314 kb)

## Data Availability

The datasets generated and/or analyzed during the current study are available from the corresponding author upon reasonable request.
