# Supplementary material for: Nomogram for Predicting Early Mortality after Umbilical Cord Blood Transplantation in Children with Inborn Errors of Immunity
Source: J Clin Immunol. 2023 May 8;43(6):1379–92. doi: 10.1007/s10875-023-01505-8 (PMC10354135; doi:10.1007/s10875-023-01505-8)
Supplement: Supplementary file 1 — (DOCX 1314 kb) [file 10875_2023_1505_MOESM1_ESM.docx]

**Table S1**. Definition of medical history

| **Variable** | **Definition** |
| --- | --- |
| Sepsis | In accordance with definitions for sepsis in pediatrics proposed by the international pediatric sepsis consensus conference [1] |
| Pneumonia | Diagnosed by two independent senior pediatricians at any time point in the patient’s medical history |
| Pulmonary fungal infection | Based on evidence of direct microscopic examination, metagenomic next generation sequencing and culture of respiratory secretions |
| Severe pneumonia | Defined as pneumonia in the need of mechanical ventilation at any time point in the patient’s medical history |
| Intestinal infection | Diagnosed by two independent senior pediatricians at any time point in the patient’s medical history |
| Urinary tract infection | Diagnosed by two independent senior pediatricians at any time point in the patient’s medical history |
| CNS infection | Diagnosed by two independent senior pediatricians at any time point in the patient’s medical history |
| SSTI | Diagnosed by two independent senior pediatricians at any time point in the patient’s medical history |
| BCG disease | The diagnostic criteria were the same as the previous study [2] |
| CMV infection | Defined as detection of CMV nucleic acid in plasma over 10^4^ copies/ml for two consecutive times at any time point in the patient’s medical history |
| EBV infection | Defined as detection of EBV nucleic acid in plasma over 10^3^ copies/ml for two consecutive times at any time point in the patient’s medical history |
| Liver dysfunction | Defined as bilirubin > 1.5× upper limit of normal (ULN), or AST/ALT> 2.5× ULN at any time point in the patient’s medical history |

BCG Bacillus Calmette-Guérin, CMV Cytomegalovirus, CNS central nervous system, EBV Epstein–Barr virus, SSTI skin/soft tissue infection, ULN upper limit of normal

**Table S2**. Clinical and biological characteristics of pediatric inborn errors of immunity (IEI) patients in training and validation cohorts.

| **Characteristics** | **Training cohort (N=152)** | **Validation cohort (N=78)** | ***p*-value** |
| --- | --- | --- | --- |
| **Demographics** |  |  |  |
| Sex |  |  | 0.764 |
| Female  Male | 31 (20.4%)  121 (79.6%) | 18 (23.1%)  60 (76.9%) |  |
| Weight (kg) | 8.5 (6.9–11.0) | 9.0 (7.5–11.3) | 0.239 |
| Height (cm) | 72 (67–84) | 75 (68–84) | 0.438 |
| BMI | 15.59 (2.35) | 15.99 (2.19) | 0.212 |
| Disease |  |  | 0.129 |
| CGD | 48 (31.6%) | 19 (24.4%) | 0.323 |
| SCID | 36 (23.7%) | 12 (15.4%) | 0.195 |
| VEO-IBD | 45 (29.6%) | 28 (35.9%) | 0.412 |
| Other IEI | 23 (15.1%) | 19 (24.4%) | 0.125 |
| Age at onset (days) | 28 (10–130) | 21 (10–91) | 0.388 |
| Age at diagnosis (months) | 7.72 (3.53–17.25) | 7.45 (3.20–18.41) | 0.978 |
| Age at UCBT (months) | 14.50 (8.67–28.38) | 14.95 (9.20–31.77) | 0.498 |
| Onset to diagnosis (months) | 4.63 (2.13–11.25) | 5.18 (1.88–15.70) | 0.464 |
| Diagnosis to UCBT (months) | 5.48 (3.11–10.24) | 7.73 (3.37–11.83) | 0.158 |
| **Medical history^*^** |  |  |  |
| Sepsis | 42 (27.6%) | 19 (24.4%) | 0.708 |
| Pneumonia | 136 (89.5%) | 63 (80.8%) | 0.104 |
| Pulmonary fungal infection | 40 (26.3%) | 19 (24.4%) | 0.871 |
| Severe pneumonia | 11 (7.2%) | 4 (5.1%) | 0.741 |
| Intestinal infection | 108 (71.1%) | 55 (70.5%) | 1 |
| Urinary tract infection | 35 (23.0%) | 15 (19.2%) | 0.622 |
| CNS infection | 7 (4.6%) | 3 (3.8%) | 1 |
| SSTI | 78 (51.3%) | 44 (56.4%) | 0.553 |
| CMV infection | 22 (14.5%) | 10 (12.8%) | 0.887 |
| EBV infection | 9 (5.9%) | 3 (3.8%) | 0.721 |
| BCG disease | 39 (25.7%) | 11 (14.1%) | 0.065 |
| Liver dysfunction | 58 (38.2%) | 29 (37.2%) | 0.999 |
| **Laboratory tests** |  |  |  |
| Albumin (g/L) | 40.1 (35.9–43.0) | 40.0 (37.0–42.5) | 0.844 |
| ALT (U/L) | 18 (11–38) | 24 (15–38) | 0.143 |

**Table S2** (continued)

| **Characteristics** | **Training cohort (N=152)** | **Validation cohort (N=78)** | ***p*-value** |
| --- | --- | --- | --- |
| Total bilirubin (μmol/L) | 4.6 (3.4–6.1) | 3.9 (3.3–5.8) | 0.101 |
| IgA (g/L) | 0.39 (0.15–1.15) | 0.27 (0.09–0.81) | **0.035** |
| IgG (g/L) | 9.12 (6.90–12.85) | 7.60 (5.43–11.20) | **0.008** |
| IgM (g/L) | 0.79 (0.38–1.48) | 0.62 (0.34–1.13) | 0.057 |
| IgE (KU/L) | 24.79 (9.93–80.76) | 25.69 (12.22–105.35) | 0.548 |
| CD19 count (/μL) | 607.01 (302.51–1091.45) | 585.99 (262.00–962.89) | 0.416 |
| CD19 ratio (%) | 20.10 (11.91–31.15) | 20.23 (12.09–29.76) | 0.937 |
| CD3 count (/μL) | 1997.5 (1150.2–2983.7) | 1662.8 (1033.5–2424.5) | 0.050 |
| CD3 ratio (%) | 63.71 (51.89–72.72) | 60.84 (48.22–70.29) | 0.553 |
| CD4 count (/μL) | 1068.9 (619.1–1630.2) | 955.3 (579.8–1317.8) | 0.250 |
| CD4 ratio (%) | 33.66 (24.09–42.55) | 34.55 (25.30–42.81) | 0.607 |
| CD8 count (/μL) | 671.47 (386.94–1106.49) | 541.83 (298.77–880.45) | 0.083 |
| CD8 ratio (%) | 20.88 (14.18–30.01) | 20.18 (13.43–28.64) | 0.504 |
| CD56 count (/μL) | 326.48 (165.06–571.45) | 275.69 (170.02–515.87) | 0.457 |
| CD56 ratio (%) | 10.70 (5.82–18.13) | 10.99 (6.56–18.61) | 0.610 |
| elevated CRP (>= 8 mg/L) | 42 (27.63%) | 11 (14.1%) | **0.032** |
| elevated PCT (>= 0.5 ng/ml) | 2 (1.32%) | 0 (0%) | 0.789 |
| IL-6 (imputed) (pg/ml) | 26.23 (8.68–49.77) | 9.47 (2.987–64.377) | **0.004** |
| IL-6 (pg/ml) | 26.90 (12.03–51.50) | 9.47 (2.987–64.377) | **0.001** |
| ferritin (imputed) (ng/ml) | 70.08 (37.04–150.90) | 69.12 (32.19–120.65) | 0.415 |
| ferritin (ng/ml) | 70.25 (36.98–140.70) | 67.10 (31.61–112.75) | 0.332 |
| **UCB parameters** |  |  |  |
| CD34 (×10^5^/kg) | 3.43 (2.58–5.90) | 2.69 (1.85–4.80) | **0.016** |
| TNC (×10^7^/kg) | 13.23 (9.60–16.71) | 11.18 (8.32–15.16) | 0.069 |
| HLA Compatibility (/10) | 8 (8–9) | 9 (8–9) | 0.126 |
| **Early mortality** | 30 (19.7%) | 13 (16.7%) | 0.699 |

Continuous variables are presented as median (interquartile range), and categorical variables are presented as counts (percentages).

Body Mass Index (BMI) is the only variable that follows a normal distribution; thus, it is presented as mean (SD).

^#^Since thorough eradication of residual infection in patients with IEI is particularly difficult and there is a lack of uniformity in the definition of ‘active infection’, ‘medical history’ represents ever had infection or active infection.

ALT alanine aminotransferase, BCG Bacillus Calmette-Guérin, BMI body mass index, CGD chronic granulomatous disease, CMV Cytomegalovirus, CNS central nervous system, EBV Epstein–Barr virus, HLA human leucocyte antigen, VEO-IBD: very early onset inflammatory bowel disease, SCID severe combined immunodeficiency, SSTI skin/soft tissue infection, TNC total nucleated cells, UCB umbilical cord blood

**Table S3.** Details of the genetic diagnosis of the patients

| **Disease** | **Genetic defect** | **N (%)** |
| --- | --- | --- |
| **SCID** |  | **48 (20.87%)** |
| SCID(T-B+) | *IL2RG* | 15 (6.52%) |
|  | *JAK3* | 5 (2.17%) |
|  | *CD3E* | 1 (0.43%) |
|  | *IL7R* | 1 (0.43%) |
| SCID(T-B-) | *RAG1* | 11 (4.78%) |
|  | *RAG2* | 6 (2.61%) |
|  | *ADA* | 4 (1.74%) |
|  | *DCLRE1C* | 1 (0.43%) |
|  | *LIG4* | 2 (0.87%) |
|  | Unspecified | 1 (0.43%) |
| MHC class II deficiency* | *CIITA* | 1 (0.43%) |
| **CGD** |  | **67 (29.13%)** |
| X-linked CGD | *CYBB* | 60 (26.09%) |
| Autosomal recessive CGD | *CYBA* | 3 (1.30%) |
|  | *NCF1* | 1 (0.43%) |
|  | *NCF2* | 3 (1.30%) |
| **VEO-IBD** |  | **73 (31.74%)** |
| IL-10R deficiency | *IL10RA* | 72 (31.3%) |
|  | *IL10RB* | 1 (0.43%) |
| **Other IEI** |  | **42 (18.26%)** |
| CD40 ligand deficiency | *CD40LG* | 9 (3.91%) |
| DOCK8 deficiency | *DOCK8* | 3 (1.30%) |
| ZAP-70 deficiency | *ZAP70* | 1 (0.43%) |
| WAS | *WAS* | 9 (3.91%) |
| STAT3 deficiency | *STAT3* | 1 (0.43%) |
| CARD11 deficiency | *CARD11* | 1 (0.43%) |
| STAT5b deficiency | *STAT5B* | 1 (0.43%) |
| TRNT1 deficiency | *TRNT1* | 1 (0.43%) |
| IPEX | *FOXP3* | 2 (0.87%) |
| CD25 deficiency | *IL2RA* | 1 (0.43%) |
| LRBA deficiency | *LRBA* | 3 (1.30%) |
| SAP deficiency (XLP1) | *SH2D1A* | 1 (0.43%) |
| XIAP deficiency (XLP2) | *XIAP* | 1 (0.43%) |
| Severe congenital neutropenia | *ELANE* | 1 (0.43%) |
| Leukocyte adhesion deficiency type 1 | *ITGB2* | 6 (2.61%) |
| CINCA | *NLRP3* | 1 (0.43%) |

*One patient with major histocompatibility complex (MHC) class II deficiency in our cohort presented with a clinical phenotype (postnatal recurrent infection, severe pneumonia requiring mechanical ventilation, failure to thrive, and opportunistic infections) and immunophenotype (decreased T cell and B cell deficiency) that were very similar to SCID, hence this patient was included in the SCID group.

CGD chronic granulomatous disease, CINCA chronic infantile neurologic cutaneous and articular syndrome, IPEX immune dysregulation, polyendocrinopathy, enteropathy X-linked, SCID severe combined immunodeficiency, WAS Wiskott-Aldrich Syndrome, XLP X-linked lymphoproliferative disease, VEO-IBD very early onset inflammatory bowel disease

**Table S4.** Comparison of performances of multivariate logistic regression models with different features sets.

| **NO** | **Formula** | **AIC**  **(model fitting on training set)** | **AUC (training set)** | **Bootstrap-corrected AUC (training set)** | **AUC**  **(temporal validation set)** | **Notes** |
| --- | --- | --- | --- | --- | --- | --- |
| 1 | survive ~ Alb | 135.72 | 0.7458  [0.6372, 0.8543] | 0.7459 | 0.6237  [0.4258, 0.8215] |  |
| 2 | survive ~ Alb + cd4_abs | 131.82 | 0.7792  [0.687, 0.8715] | 0.7698 | 0.6402  [0.4667, 0.8138] |  |
| 3 | survive ~ Alb + cd4_abs + elevatedCRP | 130 | 0.8003  [0.7139, 0.8866] | 0.7823 | 0.6509  [0.4734, 0.8284] |  |
| **4** | **survive ~ Alb + cd4_abs + elevatedCRP + sepsis** | **122.44** | **0.8270 [0.7409, 0.9132]** | **0.8052** | **0.7385**  **[0.5824, 0.8945]** | **Smallest AIC value** |
| 5 | survive ~ Alb + cd4_abs + elevatedCRP + sepsis + cd19_abs | 123.9 | 0.8281 [0.7433, 0.913] | 0.7998 | 0.7443  [0.5963, 0.8925] |  |
| 6 | survive ~ Alb + cd4_abs + elevatedCRP + sepsis + cd19_abs + cd4_ratio | 125.41 | 0.8306 [0.7457, 0.9155] | 0.7971 | 0.7408  [0.5833, 0.8984] |  |
| 7 | survive ~ Alb + cd4_abs + elevatedCRP + sepsis + cd19_abs + cd4_ratio + cd3_abs | 127.4 | 0.8301 [0.7449, 0.9152] | 0.7917 | 0.7408  [0.5834, 0.8983] |  |

**Table S5**. Comparison of performances of different models

| **Model category** | **Machine learning algorithms** | **Features** | **AUC**  **(training set)** | **AUC (temporal validation set)** | **Notes** |
| --- | --- | --- | --- | --- | --- |
| Linear | Multivariate logistic regression | All features (49) | 1  [1,1] | 0.5491  [0.3894, 0.7088] | *** |
|  |  | **Selected features (4)** | **0.8270**  **[0.7409, 0.9132]** | **0.7385**  **[0.5824, 0.8945]** |  |
|  | Lasso | All features (49) | 0.9014  [0.8364, 0.9663] | 0.6947  [0.5288, 0.8605] | best lambda: 0.02228786 |
| Non-linear | Random forest | All features (49) | 1  [1,1] | 0.6131  [0.4229, 0.8031] | OOB error rate: 18.42% |
|  |  | Selected features (4) | 1  [1,1] | 0.6822  [0.5027, 0.8618] | OOB error rate: 21.71% |
|  | XGBoost | All features（49） | 0.9669  [0.9296, 1] | 0.6627  [0.4786, 0.8468] |  |
|  |  | Selected features (4) | 0.9335  [0.8831, 0.9839] | 0.6888  [0.5105, 0.867] |  |

*glm.fit: algorithm did not converge

*glm.fit: fitted probabilities numerically 0 or 1 occurred; prediction from a rank-deficient fit may be misleading.

OOB: out of bag

**Table S6**. Performances of the optimum performing model with disease information

| **NO** | **Formula** | **AUC (training set)** | **Bootstrap corrected AUC**  **(training set)** | **AUC (temporal validation set)** | **Notes** |
| --- | --- | --- | --- | --- | --- |
| **1** | **survive ~ Alb + cd4_abs + elevatedCRP + sepsis** | **0.8270**  **[0.7409, 0.9132]** | **0.8052** | **0.7385 [0.5824, 0.8945]** | **Final best-performing model** |
| 2 | survive ~ Alb + cd4_abs + elevatedCRP + sepsis + SCID | 0.8257  [0.74, 0.9114] | 0.7990 | 0.7373 [0.5805, 0.894] | + SCID (0/1) |
| 3 | survive ~ Alb + cd4_abs + elevatedCRP + sepsis + CGD | 0.8260  [0.7372, 0.9147] | 0.7989 | 0.7219  [0.5624, 0.8814] | +CGD |
| 4 | survive ~ Alb + cd4_abs + elevatedCRP + sepsis + VEOIBD | 0.8265  [0.7408, 0.9122] | 0.7999 | 0.7349 [0.5767, 0.8931] | +VEOIBD |
| 5 | survive ~ Alb + cd4_abs + elevatedCRP + sepsis + SCID + VEOIBD | 0.8265  [0.7409, 0.9121] | 0.7903 | 0.7349 [0.5768, 0.893] | +SCID, VEOIBD |
| 6 | survive ~ Alb + cd4_abs + elevatedCRP + sepsis + SCID + CGD | 0.8262  [0.7377, 0.9148] | 0.7870 | 0.7219 [0.5624, 0.8814] | +SCID, CGD |
| 7 | survive ~ Alb + cd4_abs + elevatedCRP + sepsis + CGD + VEOIBD | 0.8238  [0.7345, 0.913] | 0.7891 | 0.7266 [0.5646, 0.8887] | +CGD, VEOIBD |
| 8 | survive ~ Alb + cd4_abs + elevatedCRP + sepsis + SCID + CGD + VEOIBD | 0.8246  [0.7337, 0.9155] | 0.7799 | 0.7183 [0.5559, 0.8808] | +SCID, CGD, VEOIBD |


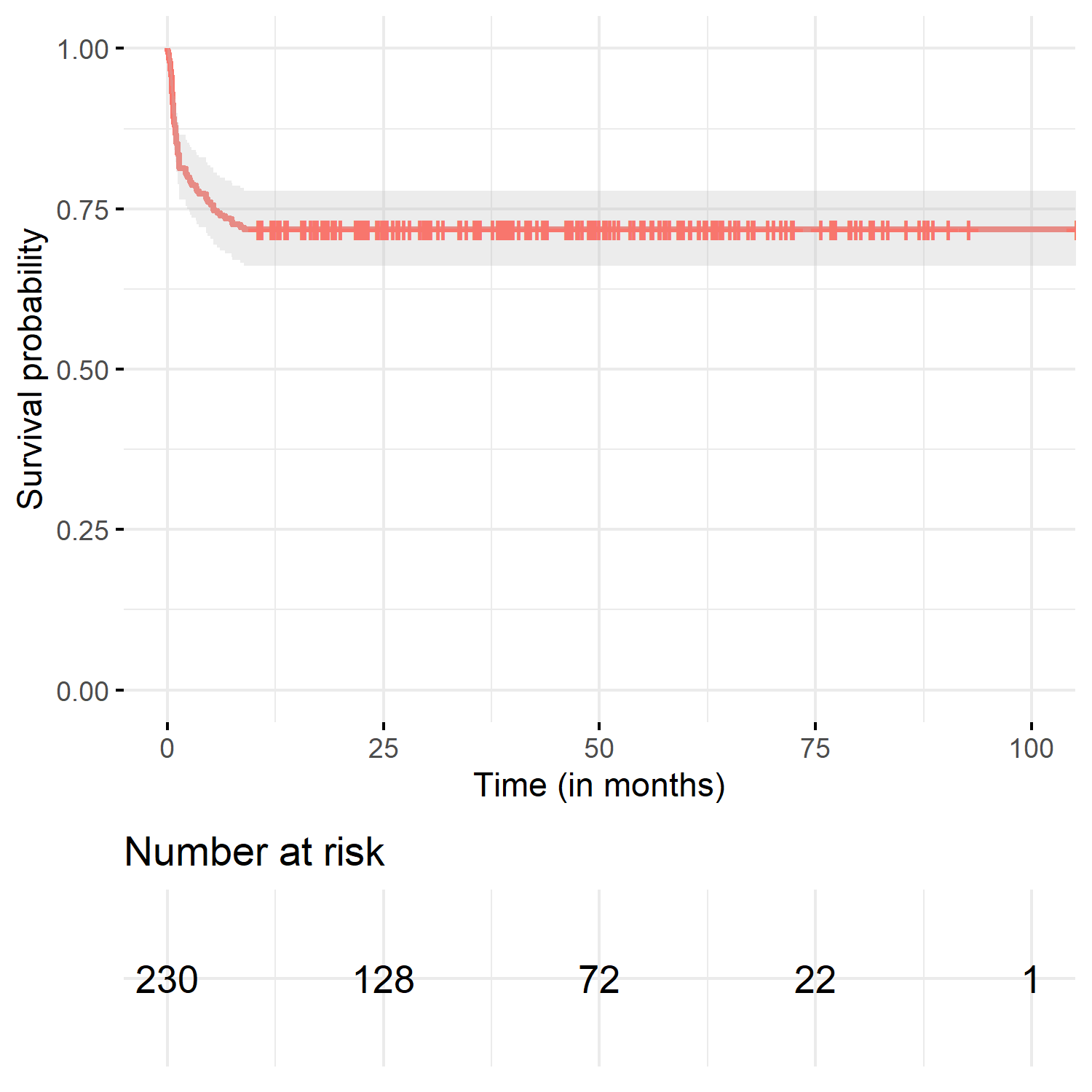


**Fig. S1** Overall survival of all 230 patients (overall survival rate: 71.7%).

**
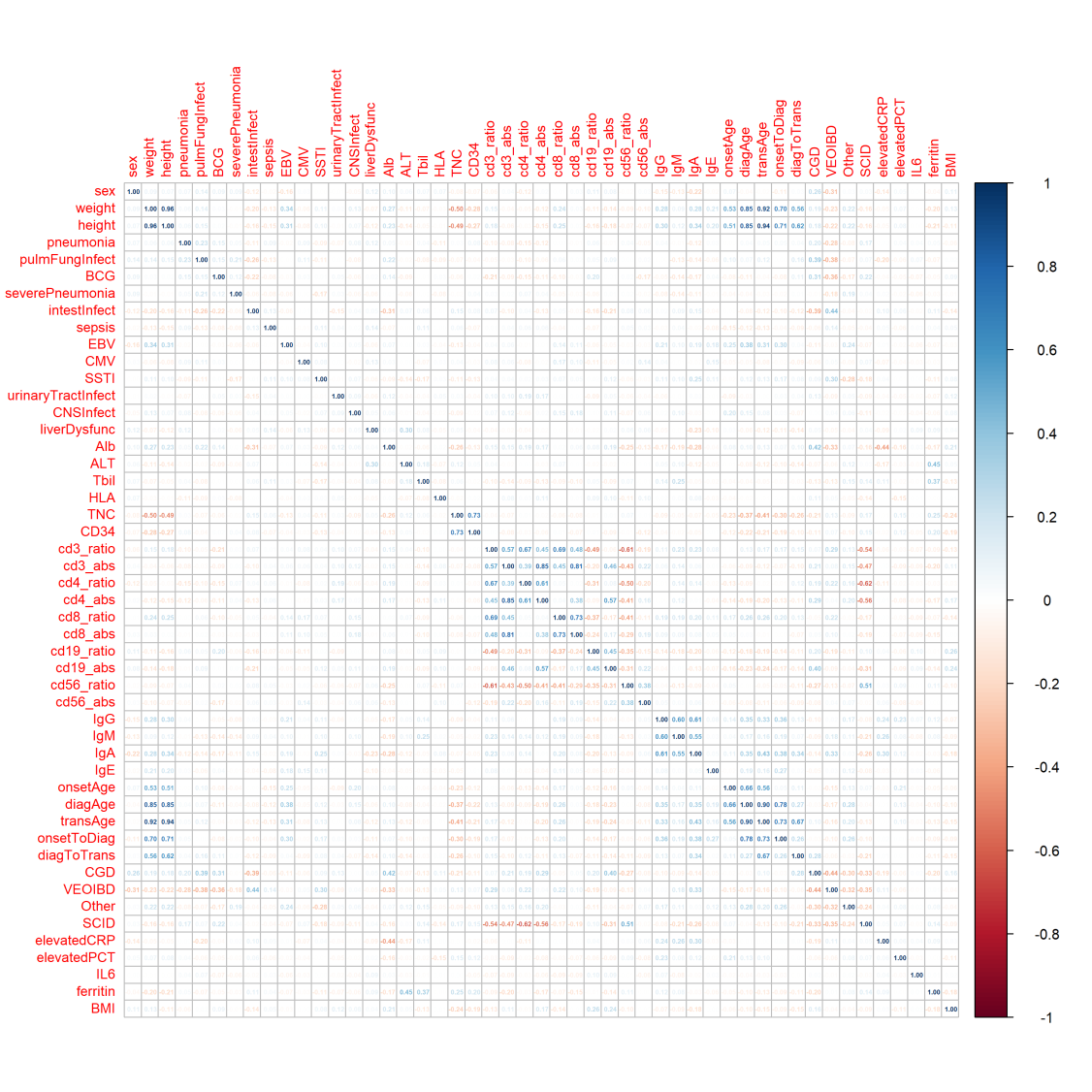
**

**Fig. S2** Heat map of the correlation matrix to measure predictive variables’ collinearity

Each cell is filled with a number and is represented by a color. The number indicates the correlation coefficient between the two corresponding variables across all patients. No perfect collinearity was found in the data matrix.

**
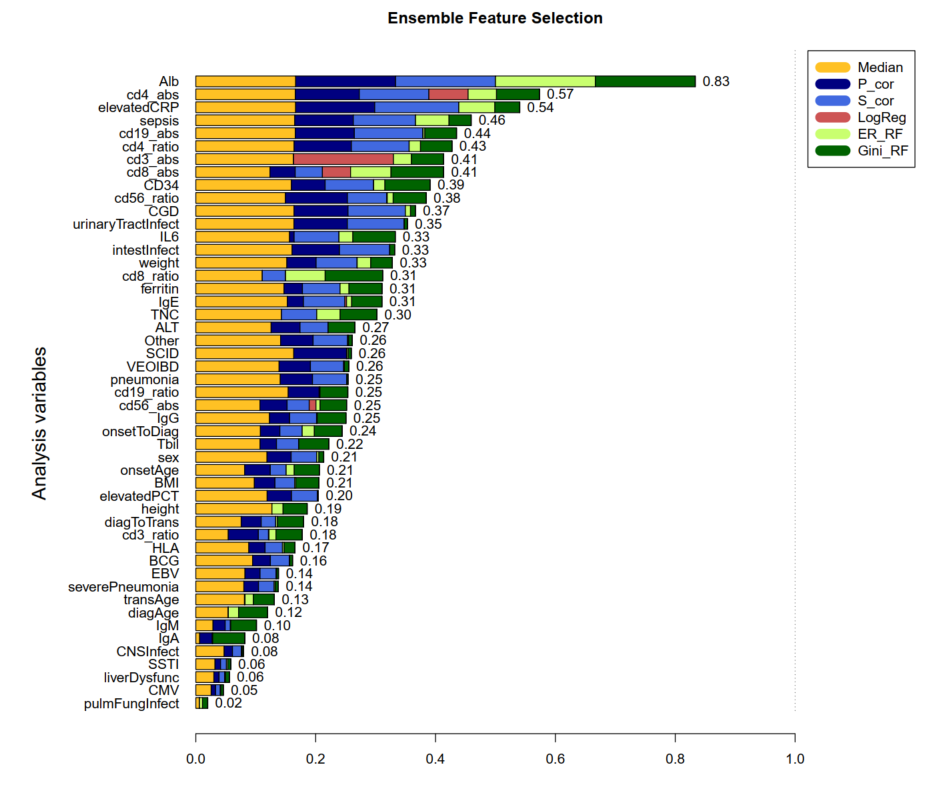
**

**Fig. S3** Variable ranking based on ensemble feature selection score

The y-axis shows the 49 variables ordered by importance value. The x-axis shows the cumulative importance values, calculated via an ensemble of feature selection methods including Median, Pearson’s product moment correlation test (P_cor), Spearman’s rank correlation test (S_cor), beta-values of logistic regression (Log_Reg), error-rate-based variable importance measure (ER_RF), and Gini-index-based variable importance measure (Gini_RF).

**A**

**
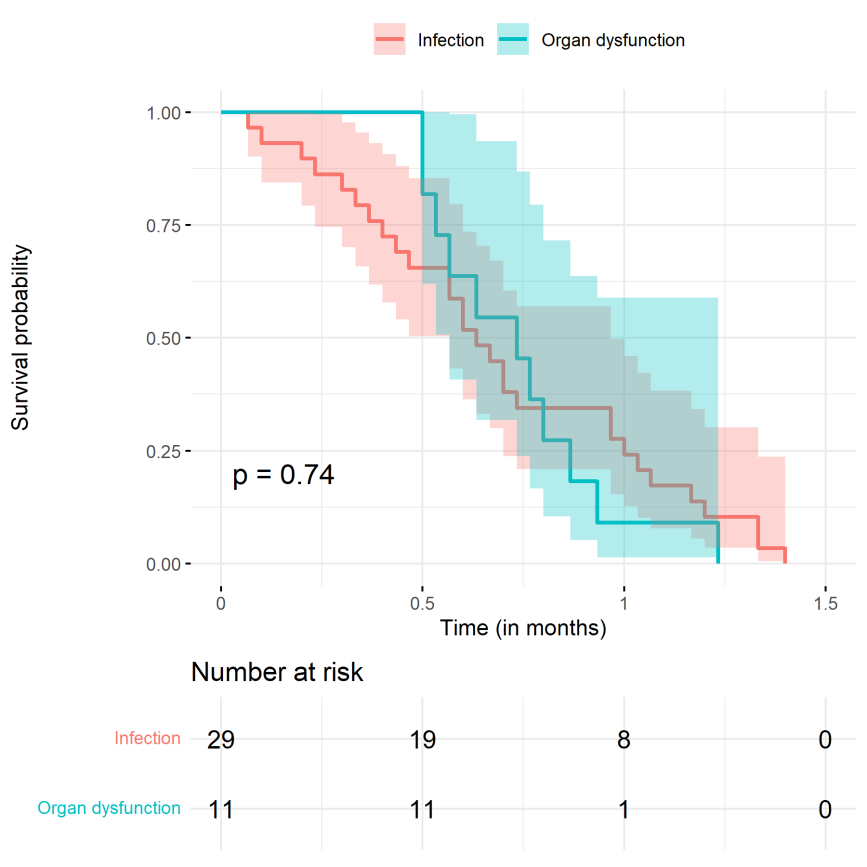
**

**B**

**
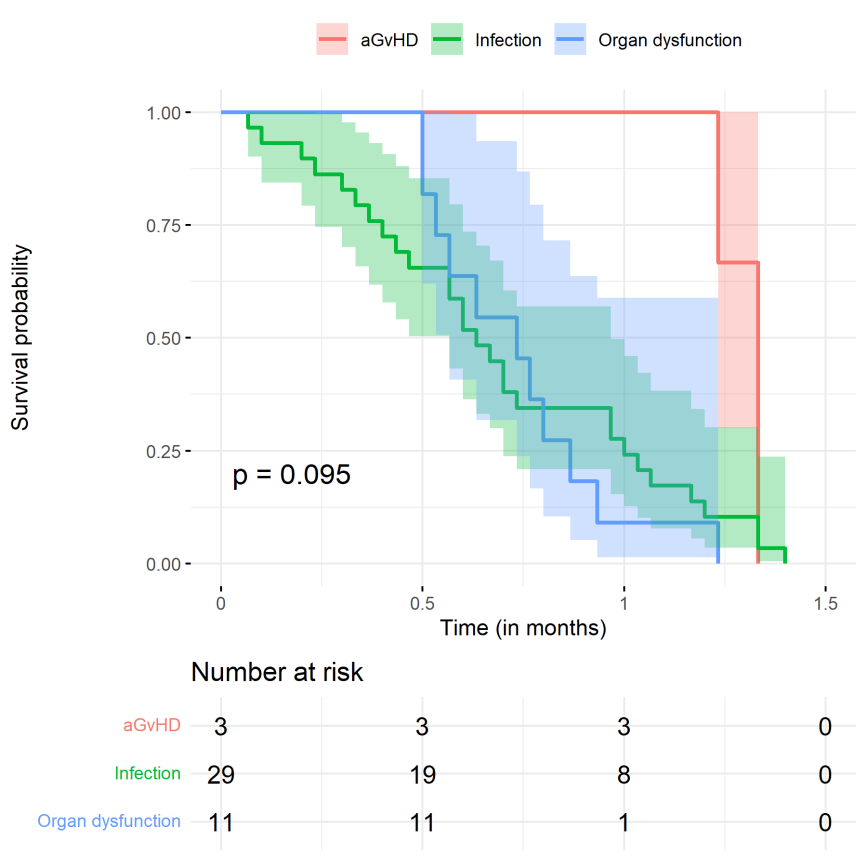
**

**Fig. S4** Kaplan–Meier (KM) survival curves for patients with different death causes

A. The two survival curves (infection vs. organ dysfunction) were compared by log-rank test and there was insignificant difference between the two curves (p = 0.74).

B. The three survival curves (infection, organ dysfunction and aGvHD) were compared by log-rank test and there was insignificant difference between the two curves (p = 0.095).

**Supplementary Reference**

1. Goldstein B, Giroir B, Randolph A. International pediatric sepsis consensus conference: definitions for sepsis and organ dysfunction in pediatrics. Pediatric critical care medicine. 2005;6(1):2-8. <https://doi.org/10.1097/01.PCC.0000149131.72248.E6>
2. Zeng Y, Ying W, Wang W, et al. Clinical and Genetic Characteristics of BCG Disease in Chinese Children: a Retrospective Study [published online ahead of print, 2023 Jan 20]. J Clin Immunol. 2023;10.1007/s10875-022-01422-2. doi:10.1007/s10875-022-01422-2
